# ADCC: the rock band led by therapeutic antibodies, tumor and immune cells

**DOI:** 10.3389/fimmu.2025.1548292

**Published:** 2025-04-16

**Authors:** Roos Vincken, Uxue Armendáriz-Martínez, Ana Ruiz-Sáenz

**Affiliations:** ^1^ Department of Cell Biology, Erasmus University Medical Center Rotterdam, CN, Rotterdam, Netherlands; ^2^ Center for Cooperative Research in Biosciences (CIC bioGUNE), Basque Research and Technology Alliance (BRTA), Bizkaia Technology Park, Derio, Spain; ^3^ IKERBASQUE, Basque Foundation for Science, Bilbao, Spain

**Keywords:** antibody-dependent cellular cytotoxicity, cancer immune co-cultures, natural killer cells, ADCC readouts, therapeutic antibodies, immunotherapy

## Abstract

Antibody-dependent cellular cytotoxicity (ADCC) is a critical mechanism by which therapeutic antibodies leverage the immune system to target and eliminate cancer cells. The key agents of ADCC are natural killer (NK) cells, specifically targeting antibody-covered cancer cells through the CD16 receptor. While other immune cells and Fc receptors can contribute and enhance ADCC, NK cells and the CD16 receptor are crucial for the efficacy of cancer therapies such as trastuzumab, cetuximab and rituximab. Co-culture assays are essential for understanding the mechanisms of these therapies, overcoming resistance and optimizing novel therapeutic antibodies. This review highlights the importance of measuring ADCC to assess the efficacy of therapeutic antibodies. Here we also present the various *in vitro* models and assay methodologies available for studying ADCC, comparing the strengths and limitations of approaches like using PBMCs to better reflect real-life conditions or NK cell lines for standardization. It also covers different readouts for ADCC, either focusing on effector cells activation, including reporter and degranulation assays or in the target cell killing, including different molecule release assays, flow cytometry and immunofluorescence techniques. Selecting the best model for studying ADCC is crucial for the translational significance of therapeutic antibody research.

## Introduction

Therapeutic antibodies have revolutionized the field of cancer therapy. These agents can engage the immune system to attack the cancer cells through antibody-dependent cellular cytotoxicity (ADCC). These antibodies are designed to recognize cancer-specific epitopes or targets overexpressed by the cancer cells. Many of these antibodies require Fc-FcγR interactions for their antitumor activity (1). Upon binding to cancer cells via the fab region, the Fc domain of these antibodies engages FcγR on immune cells. This interaction triggers the activation of signaling pathways in immune cells that result in release of cytotoxic vesicles containing perforin and granzymes to induce cell death in the targeted cancer cells. Using this mechanism, we can direct the immune system specifically towards the cancer.

One such therapy is trastuzumab, a monoclonal antibody targeting the extracellular domain of HER2. Trastuzumab is the first-line treatment for HER2-postive (HER2+) breast cancer and has been leading the ADCC rock band for over 20 years (2–4). With trastuzumab leading the way, over 40 other antibody-based therapies (5) have been approved in the clinic (6, 7). Many of these antibody-based targeting therapies such as cetuximab exert their function by inhibiting specific signaling pathways (8) and some of them have been further improved by attaching a cytotoxic payload that is internalized into the cancer cell upon antibody binding. It is worth noting that these antibody-drug conjugates such as trastuzumab-emtansine (T-DM1) and trastuzumab-deruxtecan (T-DXd) retain the ability of trastuzumab of triggering ADCC, combining this immune-mediated mechanism with the delivery of potent cytotoxic agents for enhanced antitumor efficacy (9, 10).

Not all therapeutic antibodies target tumor cells. Immune checkpoint inhibitors, for instance, modulate the immune system to enhance anti-tumor responses. The role of ADCC in these therapies remains unclear. In some cases, antibodies are designed to deliberately eliminate ADCC to prevent adverse effects (11). However, in others such as anti-CTLA-4 antibodies, which block T-cell inhibition during antigen presentation, ADCC may play a beneficial role. While eliminating T cells is not the intended outcome, studies suggest that ADCC-mediated depletion of intratumoral regulatory T cells can enhance tumor control (12–14). This principle may also be applied to PD-1/PD-L1 inhibitors. In particular, PD-L1 blocking antibodies could improve efficacy by promoting ADCC against tumor cells (15, 16). Further research is needed to clarify the contribution of ADCC to the mechanisms of these antibodies.

Though the efficacy of these therapies depends largely on the interaction with immune cells, many *in vitro* studies studying trastuzumab efficacy and resistance rely on assays measuring proliferation or cell viability, often disregarding the immune component. This can result in discoveries *in vitro* with less translational potential (17). While proliferation assays are easier, in-depth studies of ADCC using suitable *in vitro* models provide more relevant information about the efficacy of antibody-based therapeutics revealing which factors influence the success or failure of this rock band (18–28).

## Immune cells mediating ADCC

Natural killer (NK) cells are the main agent responsible for killing cancer cells through antibody-dependent cellular cytotoxicity. NK cells are generally characterized as CD3 negative CD56 positive cells (29) and comprise 5 to 20% of the peripheral blood mononuclear cells (PBMCs) (30). Two subsets of NK cells have been described which express different cell surface molecules and are functionally distinct. 95% of the NK cells in the blood are CD56^dim^CD16^+^. This subset is highly cytotoxic and due to its expression of CD16 it is capable of antibody recognition and is the primary mediator of ADCC. The other CD56^bright^CD16^-^ subset is mainly present in the lymph nodes and, while capable of cytokine production, only exerts low cytotoxicity (31).

Patient studies have provided evidence for the role of NK cells in therapeutic antibody efficacy. In HER2+ breast cancer patients, infiltration of NK cells is increased in response to neoadjuvant trastuzumab treatment, suggesting their involvement in trastuzumab action (32). In addition, studies isolating NK cells from patients show that those with higher levels of NK cells have a longer progression free survival (PFS) (33), while the patients with no *in vitro* ADCC results did not respond to treatment (34). This correlation is not only confined to breast cancer, as NK cell activity is also associated with relapse-free survival of colorectal cancer patients treated with cetuximab (35).

*In vitro* assays have been employed to separately study the contribution of the different immune cell subsets to ADCC. Kute et al., demonstrated *in vitro* that NK cells are twice more cytotoxic against HER2+ breast cancer cells than monocytes (36). While the cell killing mediated by NK cells is almost exclusively reliant on trastuzumab, the monocyte-mediated killing is predominantly antibody independent. Regarding macrophages, addition of trastuzumab increases cancer cell killing by these immune cells *in vitro* through antibody-dependent phagocytosis (37). In accordance with Kute et al, less than 50% of the cell killing mediated by the macrophages is trastuzumab dependent. In the case of B and T lymphocytes, they also show low levels of cytotoxicity, though partially mediated by NK contamination (36). Several studies have identified a specific subset of γδ T cells capable of ADCC. Infusions of this subset in mice models resulted in an increase in trastuzumab efficiency (38). Although this T cell subset offers an opportunity for immune therapy, it is unlikely to be a main contributor to trastuzumab efficacy (39).

Further evidence of the significance of NK cells in ADCC is provided by studies depleting specific immune cell subsets from the PBMC population before co-culturing with target cells. While the depletion of monocytes does not significantly affect cytotoxicity of the PBMCs (40), the depletion of NK cells reduces or eliminates antibody-mediated cellular cytotoxicity *in vitro* (40–42) and in mouse models (43, 44).

## Immune receptors mediating ADCC

ADCC performing immune cells are activated through the signaling of Fc gamma receptors (FcγR). These receptors specifically bind to the Fc domain of the antibodies coating the target cells (45) and are crucial for linking antibody-mediated immune responses to cellular effector functions such as ADCC, phagocytosis and immune regulation. The main Fc receptors that bind IgG antibodies are FcγRI (CD64), FcγRIIa (CD32A), FcγRIIb (CD32B), FcγRIIc (CD32C), FcγRIIIa (CD16A) and FcγRIIIb (CD16B). Primarily they are activating receptors, only FcγRIIb inhibits activation of immune cells to prevent excessive inflammation (46).

FcγRI binds monomeric IgG molecules and is expressed on phagocytotic cells. Out of all Fc receptors the FcγRI has the highest affinity, but its constitutive saturation attenuates its function. The FcγRIIIb receptor is expressed in high amounts by neutrophils. However, this receptor lacks internal signaling motifs and acts as decoy receptor. FcγRIIc can enhance ADCC but is functionally inactive in 80% of humans due to a premature stop codon. The FcγRIIIa and FcγRIIa activating receptors along with the inhibitory FcγRIIb receptor, participate in antibody-dependent cellular phagocytosis (ADCP), antibody-dependent cellular cytotoxicity (ADCC), as well as in the induction of cytokines and chemokines. FcγRIIa and FcγRIIIa are both present in monocytes, macrophages and dendritic cells. In these subsets of immune cells antibody-mediated activation is regulated by the balance between activating and inhibitory FcγRs. In contrast, NK cells predominantly express the activating receptor FcγRIIIa and are not inhibited by FcγRIIb (46).

More details about these receptors such as their structure, domains, genetic variability, affinity and functions have been previously extensively reviewed (47–49).

### CD16, a mayor player

The strength of ADCC is influenced by several factors, including IgG subclass, glycosylation and antibody polymorphisms (45), but also by polymorphisms in the Fc receptors recognizing the antibody. Polymorphisms in these Fc receptors generate variants that bind to the Fc region of antibodies with different affinities thereby mediating sensitivity to therapy. HER2+ breast cancer patients with FcγRIIIa-158 V/V genotype exhibited a higher objective response rate (ORR) and a longer PFS in response to trastuzumab than patients with FcγRIIIa-158 F/V or F/F variants (50). The same relationship between FcγRIIIa polymorphisms and response to therapeutic antibodies is observed in non-Hodgkin lymphoma patients treated with rituximab (51, 52) and efficacy of cetuximab in metastatic colorectal cancer patients (53, 54).

Furthermore, PBMCs harboring FcγRIIIa and FcγRIIa high-affinity variants were also significantly associated with higher trastuzumab-mediated ADCC *in vitro*, which was even higher in PBMCs with the combination of both variants (50). However, in other studies only blocking of FCγRIIIa, but not FCγRIIa, greatly reduced ADCC of PBMCs (42), indicating a predominant role for FCγRIIIa. This receptor is also the only one capable of independently triggering cytotoxicity. Its activation can be enhanced or inhibited by costimulatory or inhibitory receptors, but their signaling is not required (55).

## Application of co-culture assays

Co-culture assays have been instrumental in identifying mechanisms of resistance and developing strategies to overcome them. Several studies have combined CRISPR screens with high-throughput ADCC assays to uncover novel mechanisms. On the immune side, these studies have identified cytokines and NK cell subsets that promote resistance in melanoma (18). On the tumor side, they revealed that loss and overexpression of certain proteins confers resistance to daratumumab for multiple myeloma (19). Hypothesis-driven studies have further identified the expression of the membrane receptor CD64 as a mediator of resistance to anti-CD123 for AML using *in vitro* ADCC assays. This discovery led to the development of a novel anti-CD123 antibody with added NKp46-engaging molecules to restore sensitivity. Consistent with the *in vitro* data, this novel antibody performed well in both mouse models and in primates and is now in the preclinical stage (20). Another example of identification of a resistance mechanism resulting in novel drug development is the role of ICAM-1 downregulation in conferring resistance to trastuzumab. This finding prompted the development of CAR-NK cells to reduce the need for ICAM-1 or other co-stimulation (21). Additionally, another study tested combination of existing antibody drugs and small molecule kinase inhibitors to overcome resistance (22).

In developing novel therapeutic antibodies and drug combinations, *in vitro* ADCC assays are essential at the preclinical evaluation and characterization stage. For antibody selection, *in vitro* ADCC assays were used to test multiple antibodies against DDK1 peptide-HLA-A2 complex for efficacy and the specificity, selecting the best one for further testing in mice (23). Comparing novel antibodies, bemarituzumab directed against FGFR2b, demonstrated higher ADCC than existing antibodies and is specific for FGFR2b (24). In another study, *in vitro* ADCC assays demonstrated that inhibiting endocytosis effectively induces ADCC for the antibodies cetuximab, trastuzumab and avelumab (25). Furthermore, *in vitro* ADCC studies were used to investigate the mechanism of amivantamab, a bispecific antibody targeting EGFR and MET, which is now approved in several countries for treatment NSCLC patients (26). These assays also tested the effect of AFM24, a bispecific antibody targeting EGFR and CD16, on different immune cells and its effectiveness against various tumor cell subsets to identify which patients can benefit (27). Similarly, the efficacy of zenocutuzumab against different breast cancer subsets was characterized, leading to a phase I/II clinical trial for NRG1-fusion cancers (28).

## Co-culture approaches

Natural killer cells activate ADCC and eradicate the cancer cells through the signaling of FcγRIIIa (CD16). Co-culture assays using both cancer cells and immune cells are used to study this ADCC *in vitro*. While cancer cells are often the primary focus as the target cells, some considerations should be taken into account regarding the choice of effector cells. PBMCs are the most frequently used immune cells for ADCC assays, as they can be isolated from donors and are not modified to adapt to laboratory culture. However, the use of PBMCs presents several challenges. First, donor blood needs to be available and the PBMCs need to be isolated using a lengthy procedure. More importantly, studies show a high batch variability depending on the donor’s health, age and unknown factors resulting in differences in number or cytotoxic potential of the NK subset (36). Part of this can be circumvented by isolating the NK cells or even the CD16-positive NK cells from the blood, but this requires more time and laborious procedures and results in a loss of cells in the process. Even separating donors based on their CD16 variants does not eliminate the high variability (56).

For those without access to donor blood or those who wish to standardize their experiments or do high-throughput cytotoxicity screens, a cell line-based system offers many advantages. A cytotoxically active NK cell line is flexible, infinitely scalable and has a high reproducibility. Many studies have used NK cell lines for measuring ADCC (56–63). The difficulty of working with cell lines lies in that few NK cell lines have been established, and most lines lose expression of the CD16 receptor in culture, impairing their ability to mediate antibody-dependent cytotoxicity (64). To bypass this limitation, stable overexpression of CD16 restores antibody recognition and antibody-dependent cellular cytotoxicity of the NK cell lines. These models have been successfully established in NK-92 (59, 60), KHYG-1 (58, 61, 62) and NKL (56) NK cell lines. With the adequate cellular model selected, attention turns to the technical readouts of the antibody-dependent cellular cytotoxicity.

## Technical readouts of ADCC

**Figure 1:**
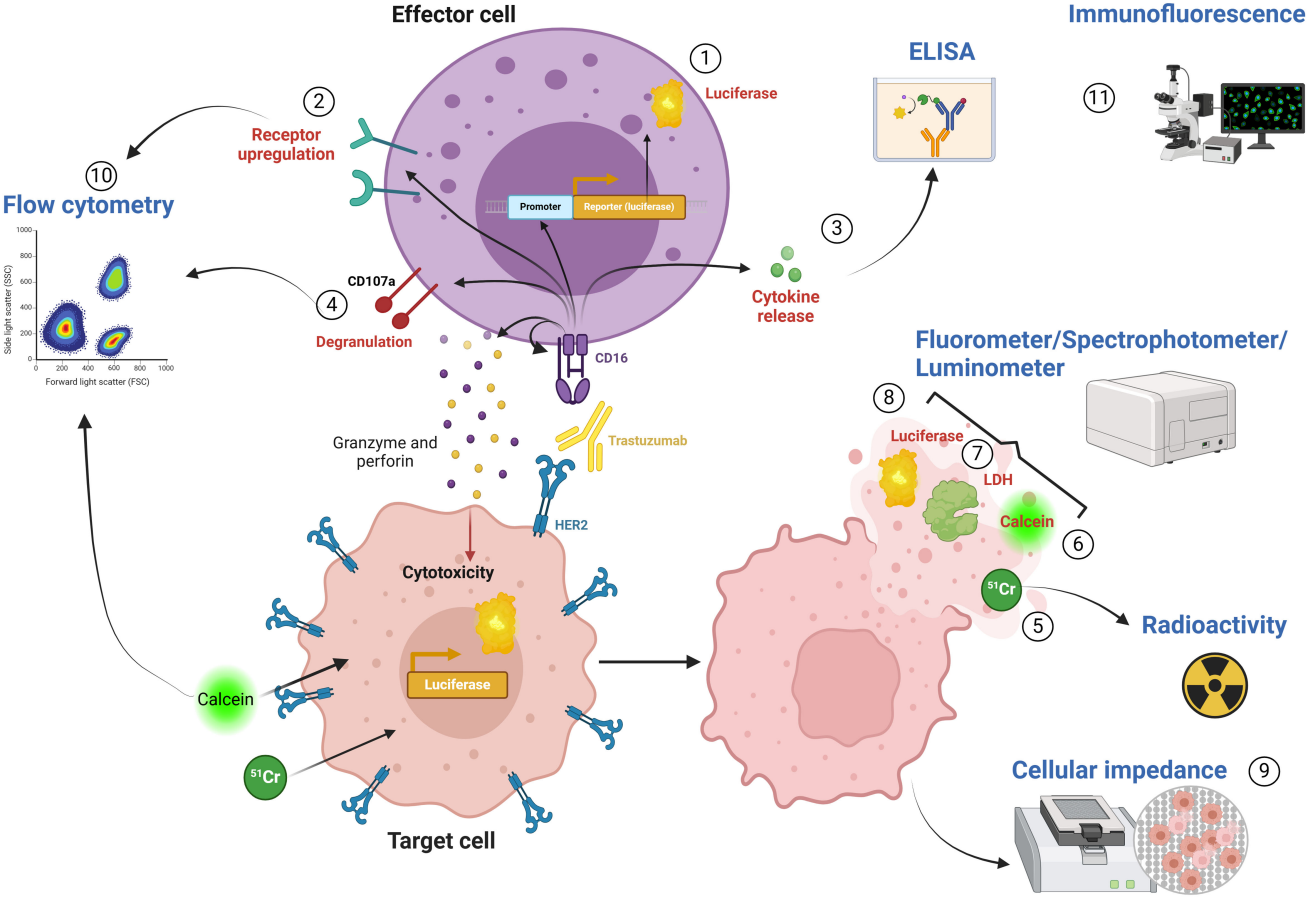
Technical readouts of ADCC. 1) Luciferase reporter assay: Luciferase expression upon CD16 activation in FcγRIIIA/NFAT-RE/luc2 engineered Jurkat T cell reporter cell line. 2) Receptor upregulation: NK cell activation can be assessed by measuring the expression of activating and inhibitory receptors on the cell surface. 3) Cytokine release: Cytokine secretion can be measured in the supernatant using ELISA assays. 4) Degranulation assay: Mobilization of receptor CD107a can be used as an indicator of granzyme- and perforin-containing vesicle release. 5) ^51^Cr-release assay: Radioactivity of ^51^Cr released into the medium can be quantified using a gamma-counter. 6) Calcein-AM assay: Released calcein can be measured via fluorometer or fluorescence retained in live cells can be measured by flow cytometry. 7) LDH release assay: LDH released by lysed target cells can be assessed by spectrophotometer. 8) Bioluminescence assay: Cytotoxicity can be measured as a decrease in bioluminescence signal upon cell death using a luminometer. 9) Cellular impedance: Cellular impedance measures the loss of impedance of the electron flow after cell lysis. 10) Flow cytometry: Flow cytometry allows several measurements including cell viability, receptor upregulation (2) and degranulation (4). 11) Immunofluorescence: Immunofluorescence allows for the visualization of cell killing by staining cells with viability or cell death stains.

In measuring ADCC, the focus can be placed on either the immune cells or the target cells. Initially, recognition of the Fc domain of the targeting antibody by CD16 leads to activation of the immune cells. This activation triggers changes in receptor expression on the immune cell surface and the release of the toxic granules which results in killing of the cancer cells. A wide array of assays is currently available to analyze the different aspects of effector activation and the final target killing (**Figure 1**, **Table 1**).

**Table 1:** Advantages (+) and disadvantages (-) of co-culture readouts.

	Immune cell			Target lysis				Combination		
	Luciferase reporter	Receptor expression	Degranulation	^51^Cr-release	Calcein-AM release	LDH release	Bioluminescence	Cellular impedance	Flow cytometry	Immunofluorescence
Target killing	––	––	––	++	++	++	++	++	++	++
Physiological relevance	––	+	+	+	+	++	+	++	++	++
Cost-effective	+	+	-	––	-/+	-	+	––	-	-
Equipment	+	-/+	-	-	+	+	++	––	-	––
Off-the shelf	++	++	-	-	-	++	––	++	-	-
Sensitivity	+	-	+	+	+	-	++	++	+	-
Multiplex	+	++	++	-	+	+	+	––	++	+
Low handling time	++	-	-	-	+	+	++	+	––	––
Safety	+	+	+	––	+	+	+	+	+	+
Standardization	++	-/+	-	+	+	+	++	++	-	-
Real-time	+	-	-	-	-	-	+	++	-	++
High-Throughput	+	+	+	-	-	+	+	+	-	––
Adherent target cells	+	+	+	+	+	+	+	+	-	+
Suspension target cells	+	+	+	+	+	+	+	-	+	-
Option to include stromal cells	+	+	+	+	+	-	+	-	+	+

Target killing: measures the killing of cancer cells (+) or indirect indicators of cytotoxicity (-); Physiological relevance: replicates the *in vivo* ADCC with NK cells (+) or uses alternative models (+); Cost-effective: relatively cheap (+) or relatively expensive (-) materials and equipment; Equipment: Technique can be performed with most standard lab equipment (+) or requires specialized equipment (-); Off-the shelf: Target or effector cells can be used directly (+) or need to be pre-labelled (-); Sensitivity: Activation or cytotoxicity is measured with high (+) or low (-) sensitivity; Multiplex: can be combined with other readouts (+) or is performed separately (-); Low handling time: Preparation is relatively easy and fast (+) or laborious (-); Safety: Technique can be safe (+) or dangerous (-) for people and the environment; Standardization: relatively easy (+) or difficult (-) to standardize between different people, laboratories or days; Realtime: Allows for readout at multiple timepoints (+) or only measures the endpoint (-); High-Throughput: Technique can be adapted for high-throughput screening (+) or is more suited for few samples (-); Adherent target cells: more (+) or less (-) suited for adherent target cells; Suspension target cells: more (+) or less (-) suited for adherent target cells; Option to include stromal cells: allows for including more cell types (+) or only a target and effector cell line (-). Dark blue color indicates a high advantage, light blue a low advantage, light orange a low disadvantage and dark orange a high disadvantage.

### NK activation

Recognition of a cancer cell by CD16 via an antibody triggers downstream signaling through the NFAT family of transcription factors. This signaling cascade forms the basis for several readouts used to measure NK cell activation.

#### Luciferase reporter

One method for measuring immune cell activation is through luciferase reporter assay. Coupling an NFAT response element to the luciferase gene results in strong luciferase expression upon CD16 activation (63). The FcγRIIIA/NFAT-RE/luc2 engineered Jurkat T cell reporter cell line is not capable of cell lysis but shows similar intracellular activation of NFAT as both primary and engineered NK cells. This cell line provides an easy and precise readout of immune cell activation. However, it is limited in physiological representation of the NK cell. Jurkat cells express a different set of receptors and though CD16 does not require co-receptors these can enhance activation. Furthermore, killing of the cancer cells is not measured by this type of assay. It can be applied for testing antibody batches, screening novel anti-CD16 monoclonal antibodies (65) and screening of engineered mAbs potency for ADCC (66).

#### Expression of receptors or cytokine release

NK cell activation also results in changes in expression of receptors on the cell membrane and release of cytokines. These changes can be used as a readout of activation without the need for an engineered reporter system. Secretion of cytokines, such as IFNγ can be measured in the supernatant using a simple ELISA assay or multiplex immunoassays such as the ProcartaPlex Luminex system (20, 21, 67). Cytokine release assays are a straightforward readout of activation of NK cells and can easily be combined with other assays using the co-culture supernatant to measure target cell lysis such as the lactate dehydrogenase (LDH) assay. Production of cytokines can also be stained intracellularly, or NK cell activation can be measured through assessing the expression of activating and inhibitory receptors on the cell surface by flow cytometry (68, 69). Typical markers expressed by target-activated NK cells are activation markers such as CD69, degranulation markers such as CD107a, key effector cytokines such as TNFα and IFNγ, and chemokines such as MIP-1α and MIP-1β. These markers are expressed in an activation-dependent manner and indicate both the type of immune response and strength of activation (20, 21).

#### Degranulation assay

One receptor expressed on activated NK cells that can be used as a more functional marker is CD107a. Mobilization of CD107a to the cell surface indicates the release of granzyme- and perforin-containing vesicles and is therefore more closely linked to cytotoxic killing of the cancer cells. This assay can be used to distinguish between cytotoxic effector cells and those exhibiting only cytokine production or other effector functions (69–72).

#### Immune synapse formation

The recognition of an antibody by NK cells triggers the clustering of receptors and the formation of an immune synapse, resulting in a strong binding between the two cells. This binding can be measured using flow cytometry by staining of both the target and immune cell and selecting events positive for both cell types (21). To better understand the formation of the immune synapse and the processes leading to NK activation the type and quantity of proteins in the synapse can be assessed. Examples include detection of F-actin at the synapse using confocal microscopy to evaluate NK cell spreading in response to target stiffness (73), and detection of ICAM-1 and CD16 clustering using TIRF microscopy (21).

### Target lysis

Activation and degranulation of NK cells may or may not lead to killing of the cancer cells, depending on the strength of the immune response and the sensitivity of the cancer cells. To assess cancer cell lysis, various readouts are available.

#### Intracellular molecule release assays

##### ^51^Cr-release

The classical way of measuring cytotoxicity is using the Chromium-51 release assay (74, 75). Target cells are pre-labeled with Na^51^CrO, which is incorporated into intracellular proteins, and co-cultured with effector cells to allow ADCC to take place. Lysis of the cancer cells releases the ^51^Cr into the medium, which is harvested and quantified using a gamma counter (76). While this method is valued for its simplicity and high sensitivity, ^51^Cr has a short half-life and can be spontaneously released by the target cells, affecting data accuracy. The main downside of this technique is the use of radioactive materials. These require special training, handling and disposal and have risks of harming both the individual and the environment. Due to these limitations, the Calcein-AM assay was proposed as a similar but safer alternative (77).

##### Calcein-AM

In the Calcein-AM assay, instead of radioactive chromium, the target cell is labeled with fluorescent calcein which can be measured rapidly and with high sensitivity in the supernatant from lysed cells. In addition, it also allows for the assessment of the cytotoxicity by measuring the fluorescence retained in live cells after quenching the signal released by lysed cells. The main advantage of this technique is that cells and supernatant can be recovered and reused for further assays (i.e. ELISA) which is not possible when using radioactive labeling. However, compared to ^51^Cr, calcein has a higher spontaneous leakage, although it is better than other fluorescent molecules (61, 69, 77, 78).

##### Lactate dehydrogenase assay

LDH release assay is based on a similar principle of release of intracellular contents during cell lysis. However, this assay measures the release of the cytoplasmic enzyme lactate dehydrogenase and does not use an exogenous dye. Unlike Calcein-AM or ^51^Cr, this method does not require pre-labelling of the target cells, thereby reducing the preparation time and cell loss, ideal for patient samples. As well as with the Calcein-AM assay, only part of the supernatant is taken for LDH measurement, and the remainder can be used for other experiments such as a cytokine release assay (60, 62, 79, 80).The principal disadvantage of this technique is spontaneous release of LDH into the supernatant. Although almost negligible for the majority of the target cells, the spontaneous LDH release can be high for the NK cells (62). This results in an increase of the background signal that must be subtracted to calculate the percentage of cytotoxicity. Thereby, LDH assay requires different background controls and is highly dependent on proper normalization of the data.

#### Bioluminescence

A cost-effective alternative for measuring cytotoxicity is the luciferase-based bioluminescence assay, which evaluates the luciferase activity within healthy cells compared to luciferase release into the supernatant upon cell lysis. Bioluminescence is produced when luciferase interacts with its substrate in the presence of ATP but ceases upon cell death. Thereby, cytotoxicity is quantified as a decrease in bioluminescence. Key advantages of this assay include high sensitivity (76), no interference from effector cells and no pre-labelling of the target cells. Therefore, this assay is well-suited for studies using unique target cell lines. Luciferase expressing target cells can also be co-cultured with other cell types (i.e. stromal cells) without confounding the results (81). Furthermore, this assay allows for time-course measurements from the same sample. The primary limitation is the need to generate stable luciferase-expressing cell lines. However, once a stable target cell line is established, these cells or surroundings can be modified to easily assess the effect of changes in the tumor cells, environment or effector cells on cytotoxicity (75, 82–84).

#### Cellular impedance

Cellular impedance assay provides a label-free, real-time approach to monitor cell killing. It measures the electron flow impeded by cells attached to the interface. Cell lysis results in a loss of impedance, which can be continuously monitored. It does however require the target cells to adhere and reach the exponential growth phase before effector cells can be added. However, growing the target cells past 100% confluence will result in cell death and therefore the time of starting the co-culture needs to be well monitored (85). While it is most suited for adherent target cells (36, 59, 86), it can be adjusted to include suspension target cells by tethering the cells to interface (87). A major drawback of this technique is that it requires expensive specialized equipment. This, however, allows for highly sensitive label-free real-time assay.

### Flow cytometry

Flow cytometry is a highly sensitive and versatile technique for analyzing single cells, capable of assessing subpopulations of both target and effector cells and simultaneously measuring cell death and receptor expression. However, flow cytometry is relatively expensive, technically demanding and challenging to standardize. The analysis is highly dependent on the staining efficiency and accurate gating of subpopulations. Additionally, distinguishing between NK cells and target cells typically requires labelling at least one subset or preferably both, to avoid cross-contamination. Incomplete staining of the effector cells can significantly affect the results, particularly at high effector:target ratios. Similarly, loss of fluorescent reporter expression in even a small subset of effector cells can hinder accurate separation.

Target cells are typically pre-labelled with a live-cell dye, while a cell death marker is used to assess cytotoxicity (70, 78, 88–93). Alternatively, target cells can be engineered to stably express a fluorescent protein, and cytotoxicity can be measured as the absence of these cells after co-culture. Flow cytometry measurement of cytotoxicity can be combined with commercials assays, like Pantoxilux (OncoImmunin^®^), identifying the enzymes responsible for cell killing through a fluorogenic substrate whose cleavage by granzyme B and caspases leads to fluorescence in dying cells (94). This assay has demonstrated that trastuzumab-mediated ADCC specific killing depends on granzyme B and caspase activity in HER2+ breast cancer cells (36).

Although flow cytometry offers a higher sensitivity than many other assays, a lower cytotoxicity is often observed (61, 91) due to loss of dead target cells in labelling and washing steps. This is especially pronounced in the case of adherent cells where the additional trypsinization step can induce further cell loss. Under optimal conditions, flow cytometry is highly sensitive, reliable for low target cell numbers (61), and cell death can be analyzed alongside markers expressed by target and immune cells. This, for instance, facilitates the identification of relationships between NK cell phenotype and the subsequent killing potential (93).

### Immunofluorescence

While methods like ELISA, luciferase and flow cytometry give precise quantitative data, immunofluorescence allows for visualization of cell killing. By staining cells with a viability dye, real-time quantification of cell death can be performed using a widefield incubator microscope like the Incucyte^®^ or a confocal microscope for more detail (70, 83). Immunofluorescence, however, is a laborious technique, difficult to standardize and requires extensive optimization of staining and analysis parameters to distinguish between variation and biological alterations. It is preferred for immobile adherent cells but has also been performed with suspension cells (83). Immunofluorescence allows for gaining insight into the underlying mechanism of cytotoxicity by quantifying effector cell recruitment, interaction time and effector activity (95).

#### 3D

Most of the current ADCC approaches are performed in 2D allowing tumor and NK cells to passively interact or are performed in semi 3D by centrifuging tumor and NK cells to form 3D aggregates. However, for physiological representation tumor cells should be grown in 3D spheroids (95–97), which makes quantifying cytotoxicity complex, time-consuming and low throughput. Although establishing spheroids before co-culture creates more variability, automated injection of spheroids greatly reduces variability and improves standardization. Furthermore, precise localization of the spheroids reduces imaging time, which can be a major difficulty in high-throughput immunofluorescence-based assays (98).

#### Microfluidics

To accurately recapitulate the *in vivo* mechanism, more stromal cells and even fluidics need to be incorporated into the ADCC model. Ayuso et al. developed a microfluidic device consisting of a hydrogel containing spheroids and lumens, which can be covered with endothelial cells to create artificial blood vessels. NK cells and therapeutic antibodies can be introduced in these vessels. The readouts of this model include time-lapse microscopy to assess antibody dynamics and NK cell dynamics, qPCR to assess receptor and chemokine expression, and microscopy to assess 3D cytotoxicity (99). Chernyavska et al. used a similar model with multiple channels that can incorporate target cells in 2D or 3D, along with antibodies and NK cells. While microscopy is the primary readout of cytotoxicity, cytokines can be measured in the medium, and cells can be extracted for gene expression analysis. This model can also be adjusted to incorporate endothelial cells to mimic blood vessels (100). With further development these models can be adapted to include additional stromal cells within the spheroids and hydrogel, thereby increasing their complexity (101).

## Discussion

Cancer-immune co-culture experiments are crucial when studying therapeutic antibodies capable of engaging the immune system. By closely replicating the *in vivo* condition, these models enhance our understanding of the mode of action, optimize drug discovery and help identify new biomarkers of therapy resistance. Especially as the focus in drug discovery shifts from antigen targeting to altering antibody interactions with the Fc receptors, co-culture systems are indispensable for testing both novel and modified antibodies, evaluating their ability to activate immune cells and induce cytotoxicity (45). Furthermore, these assays can be used to detect unwanted immunogenicity that could lead to reduced efficacy or allergic reactions, which is especially critical for emerging antibody-derived molecules such as nanobodies (87, 102). Using *in vitro* assays to assess desired and adverse reactions to novel drugs allows researchers to efficiently pre-screen antibodies, reducing reliance on animal models while saving time, cost and resources in drug discovery and optimization.

The choice of experimental model depends on the study goals. For initial screening, NK cell lines offer higher reproducibility and easier manipulation. For validation experiments, PBMCs are preferred, providing a closer representation of the *in vivo* immune responses and enabling the matching of patient target and effector cells. Multiple readouts are available focusing either on NK activation or target lysis. NK activation can be assessed through methods such as reporter, signaling or degranulation assays. However, while these methods are suitable for screening antibodies for CD16 activation, they do not capture the NK cytotoxic function of killing the cancer cell. Other factors around or within the target cells play a role in whether NK cell activation results in ADCC, which is especially relevant when studying resistance to therapy. For more translational relevance, readouts focusing on cancer cell killing, such as intracellular molecule release assays, bioluminescence, flow cytometry and impedance assays, are indispensable. Advanced techniques like immunofluorescence, and 3D tumor spheroid systems aim to further improve physiological representation.

Each method offers specific advantages, such as real-time monitoring or label-free detection, but also facing challenges like background noise or technical complexity. Despite the difficulties, use of co-culture systems is essential for refining drug discovery and tailoring therapies to overcome resistance effectively.
